# Epidemiology of noninvasive mechanical ventilation in acute respiratory failure - a retrospective population-based study

**DOI:** 10.1186/1471-227X-13-6

**Published:** 2013-04-09

**Authors:** Shihan Wang, Balwinder Singh, Lin Tian, Michelle Biehl, Ivaylo L Krastev, Marija Kojicic, Guangxi Li

**Affiliations:** 1 Department of Medicine, Guang An Men Hospital, China Academy of Chinese Medical Science, Beijing, China; 2 Department of Medicine, Division of Pulmonary and Critical Care Medicine, Mayo Clinic, 200 First Street SW, Rochester, MN 55905, USA; 3 Department of Medicine, The Institute for Pulmonary Diseases of Vojvodina, Faculty of Medicine, University of Novi Sad, Sremska Kamenica, Serbia

**Keywords:** Noninvasive mechanical ventilation, Acute respiratory failure, Epidemiology, Olmsted county, Health care delivery

## Abstract

**Background:**

Noninvasive mechanical ventilation (NIV) is a front-line therapy for the management of acute respiratory failure (ARF) in the intensive care units. However, the data on factors and outcomes associated with the use of NIV in ARF patients is lacking. Therefore, we aimed to determine the utilization of NIV for ARF in a population-based study.

**Methods:**

We conducted a populated-based retrospective cohort study, where in all consecutively admitted adults (≥18 years) with ARF from Olmsted County, Rochester, MN, at the Mayo Clinic medical and surgical ICUs, during 2006 were included. Patients without research authorization or on chronic NIV use for sleep apnea were excluded.

**Results:**

Out of 1461 Olmsted County adult residents admitted to the ICUs in 2006, 364 patients developed ARF, of which 146 patients were initiated on NIV. The median age in years was 75 (interquartile range, 60–84), 48% females and 88.7% Caucasians. Eighteen patients (12%) were on Continuous Positive Airway Pressure (CPAP) mode and 128 (88%) were on noninvasive intermittent positive-pressure ventilation (NIPPV) mode. Forty-six (10%) ARF patients were put on NIV for palliative strategy to alleviate dyspnea. Seventy-six ARF patients without treatment limitation were given a trial of NIV and 49 patients succeeded, while 27 had to be intubated. Mortality was similar between the patients initially supported with NIV versus invasive mechanical ventilation (33% vs 22%, P=0.289). In the multivariate analysis, the development of acute respiratory distress syndrome (ARDS) and higher APACHE III scores were associated with the failure of initial NIV treatment.

**Conclusions:**

Our results have important implications for a future planning of NIV in a suburban US community with high access to critical care services. The higher APACHE III scores and the development of ARDS are associated with the failure of initial NIV treatment.

## Background

Noninvasive mechanical ventilation (NIV) has been extensively used in the patients with acute respiratory failure (ARF) for more than two decades [1]. Before the start of NIV in intensive care unit (ICU) during 1990’s [2-5], most patients with ARF required endotracheal intubation and invasive mechanical ventilation (IMV), often complicated by airway injury, barotrauma, ventilation induced acute lung injury and ventilator associated pneumonia. Several clinical trials designed to test the efficacy of NIV in 1990’s showed great mortality benefit among patients with an acute exacerbation of Chronic Obstructive Lung Disease (AECOPD) [6,7] and acute cardiogenic pulmonary edema (ACPE) [8-10]. Besides the use of NIV for AECOPD and ACPE, the two major ARF etiologies, NIV also facilitates extubation and weaning in the ICUs [11,12]. NIV has also been recognized as a way to palliate patients with ARF who wish to avoid intubation. Palliative NIV can either be administered to offer a chance for survival, or to alleviate the symptoms of respiratory distress in terminally ill patients [13].

Although studies have shown the benefit of NIV in the treatment of patients with ARF, few epidemiological studies have investigated the epidemiology of NIV use in ARF among the critically ill patients. Especially, no population-based study has been performed to investigate the need for NIV in a defined community. The Olmsted County in Rochester, Minnesota, provides a unique opportunity to conduct a population-based study because of its unique demographics; relative geographic isolation and critical care services being provided only by a single tertiary care medical center [14-16]. Mayo Clinic serves as the only center capable of providing intensive care services in this county [17]. The long established Rochester Epidemiology Projects (REP) facilitates the data collection and ensures complete case capture from this county [15,16,18].

Therefore, we performed a retrospective population-based study to examine the use of NIV for ARF in the critically ill patients, in Olmsted County, Minnesota during the year of 2006.

## Methods

We conducted a population-based retrospective cohort study among consecutively admitted adult (≥ 18 years) patients with ARF at the Mayo Clinic medical and surgical ICUs in Rochester, MN, from January 1st 2006 to December 31st 2006. Olmsted county residents were identified based on the ZIP codes of their primary residence and verified with the REP database. The REP database is a medical record-linkage system, which links together the medical records of almost complete Olmsted County population, irrespective of any demographic or regional characteristics [15,16]. If a patient had multiple hospital admissions, only the first ARF episode was considered for analysis. The study protocol was approved by the Mayo Clinic Institutional Review Board. All eligible individuals who gave research authorization to review their medical records for research were included. Patients, who declined the use of their medical records for research, required invasive mechanical ventilation for less than 12 hours after surgical procedure and those who used CPAP treatment for sleep apnea were excluded.

### Data abstraction and management

Trained critical care clinical and research fellows abstracted the data from the electronic medical records (EMR) using a standardized protocol. The causes of NIV use were identified according to the standard definitions. The data on demographics, code status preferences, underlying severity of pulmonary and nonpulmonary organ dysfunctions, ventilation type and interface (noninvasive, endotracheal tube or tracheostomy) were extracted from the EMR. The Multidisciplinary Epidemiology and Translational Research in Intensive Care ICU datamart is an integrative database to extract ICU data from the hospital EMR [19]. Patient’s baseline characteristics, comorbidities and severity of illness (Acute Physiology and Chronic Health Evaluation [APACHE] III) scores, were collected from the EMR using the ICU datamart. Hemodynamic variables, fluid, drug infusion, laboratory parameters and ventilator settings were extracted from the ICU electronic database. All the relevant data from the patients’ medical records and bedside flow charts were reviewed from ICU admission to ICU discharge. The hospital mortality at discharge and hospital length of stay (LOS) was collected from the electronic database by manual chart review.

### Identification of noninvasive mechanical ventilation

The use of NIV was defined as the acute need of positive pressure ventilatory support through a tightly fitted facial or nasal mask for more than one hour. Acute respiratory failure was defined as the acute need of IMV support for more than twelve hours or NIV including CPAP for more than one hour [20]. Palliative NIV was defined as patients who themselves signed the “Do Not Intubate” form and those in whole the health care stuff considered NIV as the ceiling therapy to alleviate the symptoms of respiratory failure and offer a possible chance of survival. The success of NIV was defined as the recovery from acute respiratory without endotracheal intubation among patients with full code (without limitation of therapy).

### Outcomes

The primary outcome was to identify the incidence of use of NIV in patients with ARF and to identify the factors associated with the failure of the NIV among the same. The secondary outcome was to identify the long term survival among the patients with ARF who were on palliative NIV. The survivors after the hospital discharge were followed till the end of 2010. The death date was identified from the EMR or death registration record of Minnesota, in case of out-of-hospital deaths.

### Statistical analysis

All the continuous data was summarized as median (interquartile range [IQR]). Categorical data was summarized as counts and percentages. Age and gender-specific incidence rate (95% confidence interval [CI]) for NIV use in the ICU was calculated assuming that the entire population of Olmsted County (≥18 years) was at risk. The incidence rate was adjusted to the projected 2006 United States population (utilizing the data from the 2000 U.S. population census and calculating an expected 1.9% population growth per year). Kaplan-Meier survival analysis with log-rank test was used to assess the long term survival among the ARF patients who were initiated on palliative NIV. A univariate and multivariate logistic regression analysis was used to identify the factors associated with failure of NIV in full code patients. Significant variables on the univariate analysis (p < 0.2) were included in the multivariate logistic regression model. Stepwise forward and backward procedure was used to select variables included in the final analysis. Non-significant factors (p > 0.05) were eliminated (one at a time) until all remaining factors had a significant association with NIV failure. JMP statistical software (version 8.0, SAS, Cary, NC) was used for all the data analyses. The level of significance for all statistical tests was 2-sided, with P <0.05.

## Results

**Figure 1:**
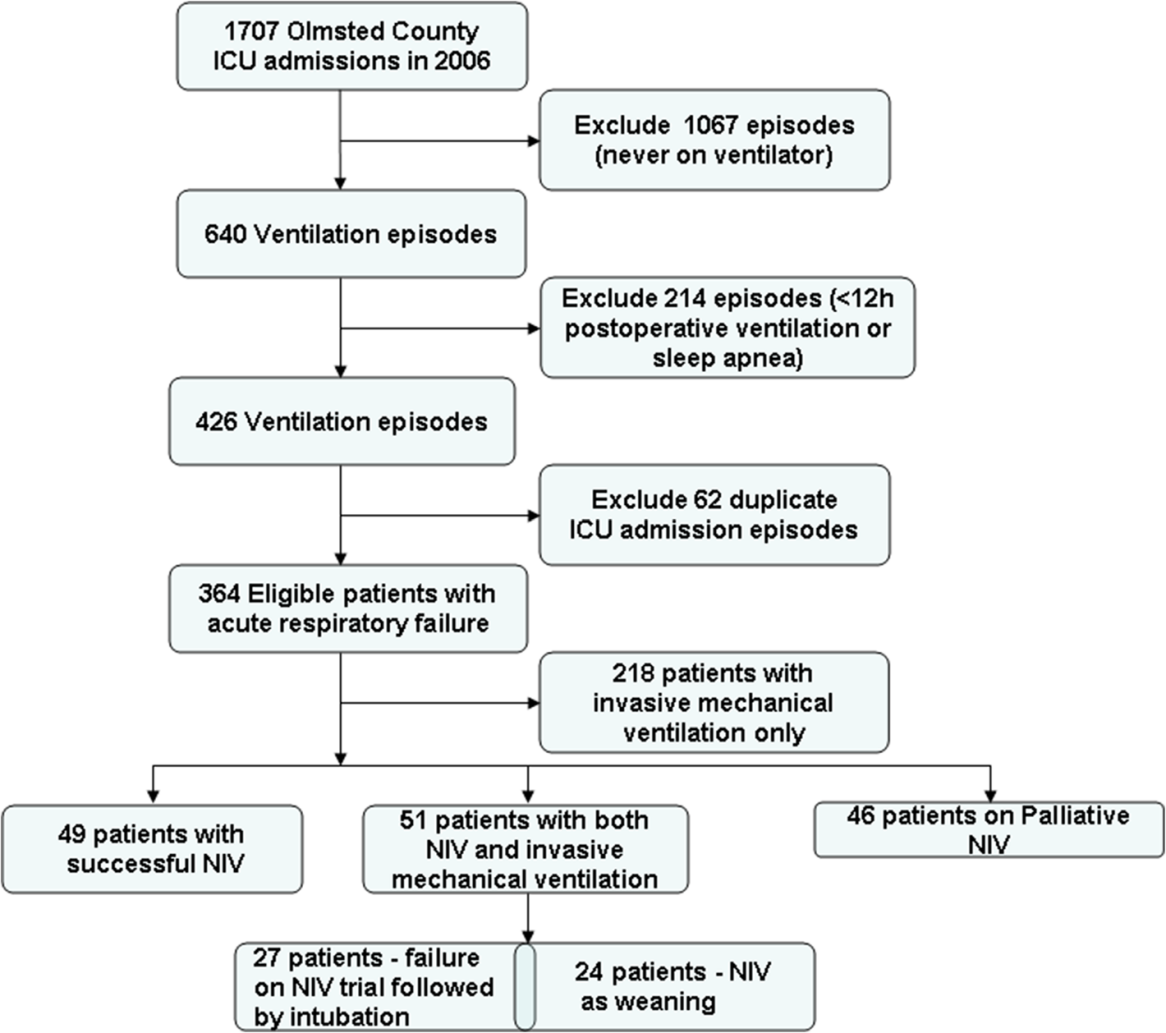
**Study outline of noninvasive mechanical ventilation use in the acute respiratory failure patients.** ICU= intensive care unit; NIV=non invasive mechanical ventilation.

In 2006, out of 1707 ICU admissions, a total of 1461 unique Olmsted County adult residents were identified. The study flowchart was shown in detail in Figure 1. Three hundred and sixty four patients developed ARF and were ventilated in ICU, among which 146 (40%) were initiated on the NIV yielding a cumulative incidence of 180 episodes per 100,000 person-years (95% CI 154~206/100,000) (Figure 1). The median age in years was 75 (IQR, 60–84), 48% were females and 88.7% were Caucasians. Eighteen patients (12%) were on CPAP mode and 128 (88%) were on NIPPV mode.

A total of 76 ARF patients were given an initial trial of NIV, out of which 49 (69%) patients succeeded, while 27 (31%) had to be intubated. The difference in the baseline characteristics, severity of illness and reasons for ARF between the patients who succeeded and failed the initial NIV trial were shown in Table 1. Patients who failed the initial NIV treatment were younger and mostly non-Caucasians. As compared to ARF patients who passed NIV trial successfully, more acute lung injury/acute respiratory distress syndrome (ALI/ARDS) and less COPD cases were present in the ARF patients who had to be intubated (Table 1). In the multivariate analysis, the development of ALI/ARDS and higher APACHE III scores were associated with the failure of initial NIV treatment (Table 2). NIV was also used in the weaning process for 24 (16%) ARF patients following IMV, of which 14 (58%) patients were re-intubated.

**Table 1:** Baseline characteristics between success and failure of initial NIV treatment

		**NIV with intubation (n=27)**	**NIV success (n=49)**	**P Value**
Age median (IQR), years		60(47–75)	70(59–82)	0.035
Female Sex, N (%)		15(56)	20(41)	0.217
Race, Caucasian (%)		20(77)	42(88)	0.002
PaO2/FiO2 ratio, Mean (SD)				
Mean PF ratio		170 (51)	192 (50)	0.088
Lowest PF ratio		91 (52)	149 (68)	<0.001
Respiratory rate, mean (SD)		20 (3)	20 (3)	0.769
APACHE III score, median (IQR)		43(33–55)	41(29–55)	0.307
Major indications for NIV, N (%)				
Acute on chronic respiratory disorders	AECOPD	1(14)	14(29)	0.007
	Asthma	0	1(2)	0.999
	Other	0	1(2)	0.999
ALI/ARDS		20(74)	4(8)	<0.001
Postoperative		1(14)	6(12)	0.999
Congestive heart failure		2(29)	10(20)	0.085
Aspiration		1(4)	1(2)	0.999
Pneumonia		0	2(4)	0.536

**Table 2:** Multivariate analysis of failure of NIV

	**Odds ratio**	**Lower 95%**	**Upper 95%**	**P Value**
Age	0.972	0.925	1.016	0.208
APACHE III Score	1.044	1.001	1.094	0.045
ALI/ARDS	33.15	7.539	205.033	<0.001
COPD	0.466	0.022	3.739	0.498
Caucasian	1.104	0.179	7.654	0.916
CHF	1.455	0.22	9.12	0.687

**Figure 2:**
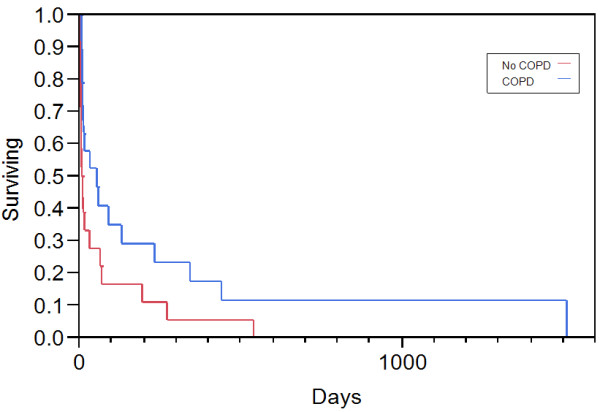
Long-term survivals between COPD and no-COPD patients on palliative NIV use COPD=Chronic Obstructive Pulmonary Disease NIV=non invasive mechanical ventilation.

Forty-six patients chose NIV as their ceiling therapy, among which 37 (10%) ARF patients were started on palliative NIV and 9 patients were initiated NIV after the withdrawal of IMV. The major etiology for those 37 patients initiated with palliative NIV was AECOPD (51%). As compared to those without COPD who were started with palliative NIV, the hospital mortality was significantly lower in the COPD patients (32% vs. 72%, p=0.01). Among the survivors, median survival time was significantly longer in patients with COPD (53 days, 95% CI 9–232) as compared to patients without COPD (8 days, 95% CI 4–30, p=0.02) (Figure 2). However, when the analysis were restricted in patients with COPD who had treatment limitation versus who did not, patients with treatment limitation had much higher hospital mortality even after adjusting for the baseline disease severity (32% vs. 0, p<0.001) (Table 3).

**Table 3:** The comparison between patients with and without treatment limitation

	**NIV with treatment limitation (n=19)**	**NIV without treatment limitation (n=25)**		**P value (treatment limitation vs. no limitation)**
		**IMV= (n=10)**	**NIV without treatment limitation (n=15)**	
Age	79(72–85)	69(67–71)	70(59–77)	0.001
Gender	10(53)	0	6(40%)	0.05
Race	16(84)	8(80)	14(93)	0.77
CAD	11(58)	6(60)	7(47)	0.76
Cancer	8(42)	3(30)	6(40)	0.76
Chemotherapy	4(21)	3(30)	1(7)	0.71
CHF	9(47)	3(30)	3(20)	0.12
DM	8(42)	3(30)	7(47)	0.99
Hypertension	17(89)	9(90)	10(67)	0.43
APACHE III score on admission	54(39–62)	45(39–63)	43(23–59)	0.14
ICU length of stay	2(1–3)	4(2–9)	1(1–3)	0.36
Hospital length of stay	6(3–9)	7(5–15)	5(4–8)	0.39
Hospital Mortality	6(32)	0	0	0.008
Median survival after Hospital discharge	43(9–232)	1341(725–2349)	1245(815–2472)	<0.001

## Discussion

In this study, we showed that NIV was commonly used in critically ill patients with ARF. NIV was used in two-third of the patients with ARF for the initial treatment and palliative care. Twenty percent of patients with ARF failed the initial trial of NIV and had to be intubated. NIV trial usually could not rescue the patients with higher severity of illness and the development of ALI/ARDS. We did not observe any significant difference in mortality between the patients who were initially supported with NIV versus IMV. Palliative NIV did not only alleviate respiratory distress but also extend the long-term survival among COPD patients who selected NIV as the ceiling therapy.

In the last two decades [21], NIV had been used extensively in the ICU settings especially among patients with ARF because of hypercapnia and cardiogenic pulmonary edema [5,6,8,11,20,22-24]. However, very few studies had discussed the use of NIV in a population-based sample [14,25]. In this population-based study, we showed that NIV was frequently used in the ICUs. NIV technique was usually performed in the ICU because of its feasibility and survival benefits [13,26,27]. In a recently published paper on the epidemiology of ARF, NIV use increased significantly from 3.8% to 10.1% [7]. In our study, we showed that NIV was used in 40% ARF patients. One of the most important reasons accounting for the higher use of NIV in our ICUs was the different method to identify ARF and NIV. In that paper, the author identified ARF cases using ICD-9 coding system, which are prone to misclassification of the ARF cases. We reviewed all the medical records to identify ARF cases and the NIV uses, which better reflect the reality of NIV practice in a suburban community tertiary medical center. Our results will be helpful in future planning for the NIV resource allocation in a community.

Although there was a significant increasing NIV use trend, it also raised the concern on the safety and potential delayed intubation. Many studies had shown that NIV should be considered as the initial treatment of ARF caused by AECOPD [5,28], ACPE [8,23,24] and other etiologies [28-30]. Our results also confirmed that AECOPD and ACPE patients could most likely succeed on the initial NIV trial. However, patients with ARDS were more likely to fail the first line NIV treatment, which support the findings of the previous studies [28,31,32]. However, the use of NIV trial on ARDS remained controversial [31-33]. A recently published randomized clinical trial showed the benefit of using initial NIV treatment in strictly selected ALI patients [34], which might not be generalizable in different ICU settings. In this study, the patients with ARF who succeeded on the NIV trials were younger and less sick (lower APACHE scores) than those who failed the NIV trials. The development of ARDS and higher APACHE III scores were strongly associated with the failure of initial NIV trials in our study, which was similar to previous observation by Rana et al. [35]. A trial of NIV in specific populations, such as ARDS patients, might potentially be harmful because it delayed the intubation and missed the best window for IMV [32,36]. However, this needs further exploration in studies specifically designed to answer this critical question. Our data did not show a significant mortality risk among patients who failed initial NIV treatment as compared to patients with initial intubation; while we did observe a trend toward the higher hospital mortality among patients who failed the initial NIV trial. Although the hospital mortality was not justified by other important confounding factors, NIV use in ARDS patients should still be cautious because the chance of success for NIV trial in this type of ARF had been very low [33,35].

Recently, Azoulay summarized ten published studies [13] and nearly half of patients on palliative NIV survived and went back home, which was similar to our findings. In the patients on palliative NIV, we also observed that around fifty percent patients survived and the median survival after hospital discharge was around 2.6 years during a four year follow-up. Certainly, the hospital mortality was significantly higher than those on IMV because of the baseline comorbidities and severity of disease. Despite an increasing use of palliative NIV, there is no evidence showing what type of respiratory failure would receive the maximum benefit from this technique. Our study did show that COPD patients might potentially get the best outcome from palliative NIV. Certainly, palliative NIV could not extend patients long term survival compared to patients without treatment limitation. The limited treatment option on NIV should not be always encouraged in COPD patients due to the worse long-term outcomes. Our findings were different from the previous report on DNI patients, wherein, they found no difference in the quality of life between the patients with and without treatment limitation and after 90 days of receiving NIV treatment for ARF [37]. Part of the reason might be related to the different study population and study design. Prospective study tended to recruit a small number of patients which might not capture the whole population on palliative care. Our study was a retrospective design and could only measure the long-term survival without the detailed information on quality of life. The population in our study was restricted to the COPD patients which limited our generalizability. Further prospective studies are needed to evaluate the benefits of palliative NIV among the critically ill patients, impact on the health economy, patient’s satisfaction and long term quality of life after hospital discharge.

Another important use of NIV was to help the intubated patients wean from IMV. Despite the decreased re-intubation rate, less complications, and better patient outcome, the role of NIV for this indication remained debatable [38]. In our primary analysis, we excluded the patients who were started on NIV after IMV because of withdrawal of care. We did not find the benefit of NIV trial on the avoidance of the re-intubation. In a recently published paper, Girault et al. [39] also showed no benefit on re-intubation rate with NIV weaning strategy. However, they found that the NIV might decrease the intubation duration and improve the weaning results in difficult-to-wean chronic hypercapnic respiratory failure patients. In spite of the frequent use of NIV in the weaning process, the evidence of NIV in these patients needs to be further investigated.

Our study had several limitations. Firstly, the retrospective observational study design raises concerns about the measured and unmeasured bias and confounding. We ensured various measures to enhance the quality of the study results, like using a standardized protocol throughout the study duration for data extraction and identification of the cases and concerning variables. Also we used the medical record linkage system to further improve the quality of the data. The other limitation of our study is the generalizibility of the results. The restricted mid-western population, predominantly Caucasian, may limit the generalizability of our study results. However, the population-based nature and various steps taken for quality assessment in our study help in addressing these concerns. Also, studies from the Olmsted County have consistently shown that their findings are generalizable to the Upper Midwest population [40], and may also provide important information regarding various diseases [16]. In addition, during the study duration, the use of NIV was not consistent on the regular hospital floors. This might slightly increase the incidence rate of NIV in ARF patients, however, it is unlikely to have affected our findings significantly.

## Conclusion

In conclusion, in this population-based study of Olmsted County residents, we showed the incidence of NIV use in patients with ARF was high and NIV was commonly used as the initial treatment strategy and for palliative care of ARF in critically ill patients. The development of ARDS and higher APACHE III score were associated with the failure of initial NIV treatment. The results of this study could be helpful in the future planning of noninvasive mechanical ventilation use in the community-based ICU settings.

## Competing interest

None of the authors have any disclosures or conflict of interest.

## Authors’ contributions

SW and GL contributed to the study design, conduct and manuscript writing. SW, BS, LT, MB, ILK and MK contributed in the data collection and the conduct of the study. WS and GL analyzed the data. SW, BS, LT, MB, ILK, MK, and GL helped with the preparation and revision of the manuscript. GL supervised and was involved as senior author in all critical parts of the study. All authors read and approved the final manuscript.

## Pre-publication history

The pre-publication history for this paper can be accessed here:

http://www.biomedcentral.com/1471-227X/13/6/prepub

## Location of study

The study was performed at Mayo Clinic, Rochester, MN, USA.
